# Cancer as a mitochondrial metabolic disease

**DOI:** 10.3389/fcell.2015.00043

**Published:** 2015-07-07

**Authors:** Thomas N. Seyfried

**Affiliations:** Biology Department, Boston CollegeChestnut Hill, MA, USA

**Keywords:** Warburg effect, fermentation, oxidative phosphorylation, mitochondria, microenvironment, cybrids, tumorigenesis, carcinogenesis

## Abstract

Cancer is widely considered a genetic disease involving nuclear mutations in oncogenes and tumor suppressor genes. This view persists despite the numerous inconsistencies associated with the somatic mutation theory. In contrast to the somatic mutation theory, emerging evidence suggests that cancer is a mitochondrial metabolic disease, according to the original theory of Otto Warburg. The findings are reviewed from nuclear cytoplasm transfer experiments that relate to the origin of cancer. The evidence from these experiments is difficult to reconcile with the somatic mutation theory, but is consistent with the notion that cancer is primarily a mitochondrial metabolic disease.

## Introduction

The prevailing view today is that cancer is a genetic disease involving nuclear mutations in oncogenes and tumor suppressor genes (Hanahan and Weinberg, 2011). A typical tumor is thought to contain two to eight so-called “driver gene” mutations that regulate the tumorigenic phenotype (Vogelstein et al., 2013; Hou and Ma, 2014). The nuclear genomic instability seen in nearly all types of tumor cells is considered the primary cause of the cancer's hallmarks that include sustained proliferative signaling, evasion of growth suppressors, resistance to cell death, replicative immortality, enhanced angiogenesis, and activation of invasion and metastasis (Hanahan and Weinberg, 2011). The somatic mutations thought to be the origin of cancer arise randomly during DNA replication in normal noncancerous stem cells (Tomasetti and Vogelstein, 2015). The somatic mutation theory reigns as the most widely accepted view of the origin of cancer and is the justification for developing personalized genetic therapies for managing the various forms of the disease (Vaux, 2011; McLeod, 2013; Hou and Ma, 2014). Despite numerous inconsistencies associated with the somatic mutation theory (Rous, 1959; Sonnenschein and Soto, 2000; Soto and Sonnenschein, 2004; Baker and Kramer, 2007; Burgio and Migliore, 2015), the theory is presented as if it were dogma in most current college textbooks of genetics, biochemistry, and cell biology, and is the mainstay of the National Cancer Institute in stating that, “*Cancer is a genetic disease—that is, it is caused by changes to genes that control the way our cells function, especially how they grow and divide*” (http://www.cancer.gov/cancertopics/what-is-cancer).

As an alternative to the somatic mutation theory, emerging evidence suggests that cancer is primarily a mitochondrial metabolic disease (Seyfried and Shelton, 2010; Hu et al., 2012; Verschoor et al., 2013; Seyfried et al., 2014). The view of cancer as a metabolic disease originated with the experiments of Otto Warburg (Warburg, 1956a,b; Burk et al., 1967). Respiratory insufficiency is the origin of cancer according to Warburg's theory. All other phenotypes of the disease, including the somatic mutations, arise either directly or indirectly from insufficient respiration (Warburg, 1956a; Seyfried, 2012a; Seyfried et al., 2014). Warburg's metabolic theory was also in line with the concepts of C. D. Darlington and others showing that cancer is largely a cytoplasmic mitochondrial disease (Woods and Du Buy, 1945; Darlington, 1948). Proponents of the somatic mutation theory, however, consider the abnormal energy metabolism of tumor cells as simply another phenotype that “*is programmed by proliferation-inducing oncogenes and defective tumor suppressor genes*” (Hanahan and Weinberg, 2011). In light of the overwhelming acceptance of the somatic mutation theory, it would be good to reconsider data from the nuclear transfer experiments that are inconsistent with the somatic mutation theory.

The rationale for the nuclear transfer experiments is to determine whether the genome of somatic cells can direct normal development (Gurdon and Wilmut, 2011). These same types of experiments can also be used to test the somatic mutation theory of cancer. If nuclear somatic mutations are the origin of cancer cells, then the hallmark cancer phenotype, *dysregulated cell proliferation*, should occur following the transfer of a tumor nucleus into a normal cell cytoplasm. In other words, the somatic mutations in the tumor cell nucleus should determine the tumorigenic phenotype of abnormal cell growth. On the other hand, if mitochondrial dysfunction is the origin of cancer cells, then the tumorigenic phenotype should follow the type of mitochondria in the cell. In other words, mitochondria from non-cancerous cells should suppress tumorigenesis whereas mitochondria of tumor cells should enhance tumorigenesis regardless of whether the nucleus present is from a normal cell or from a tumor cell. It would therefore be important to consider the findings from the nuclear-cytoplasm transfer studies, as I previously described (Seyfried, 2012d).

## Normal cytoplasm suppresses tumorigenesis in cell cybrids

Suppression of tumorigenicity was observed when the cytoplasm of enucleated normal cells was fused with nucleated tumor cells to form cybrids (Seyfried, 2012d). Cybrids contain a single nucleus with a mixture of cytoplasm from two different cells. To examine the influence of cytoplasm on the expression of tumorigenicity in cybrids, Koura fused intact B16 mouse melanoma cancer cells with cytoplasts (absent nucleus) from non-tumorigenic rat myoblasts (Koura et al., 1982). The reconstituted cybrids exhibited a unique morphology and cellular arrangements different from that of the parental cells. Tumorigenicity was reduced in all the reconstituted clones and cybrids soon after their isolation, but tumorigenicity re-appeared in some clones after extended cultivation of the cells *in vitro* (Koura et al., 1982; Seyfried, 2012d). The effects of the unnatural cell culture environment on mitochondrial respiration could account in part for the reversion to tumorigenicity seen in some clones (Warburg, 1956a; Kiebish et al., 2009). The Koura et al findings showed that normal cytoplasm, containing mitochondria from non-tumorigenic cells, could suppress the malignant phenotype of tumor cells (Seyfried, 2012d). Although these observations were not linked to Warburg's theory, the findings question the dominant role of the nucleus in the origin of tumorigenesis.

In a more comprehensive series of experiments, Israel, and Schaeffer demonstrated that suppression of malignancy could reach 100% in cybrids containing tumorigenic nuclei and normal cytoplasm (Israel and Schaeffer, 1987). On the other hand, tumors formed in 97% of mice implanted with cybrid cells derived by fusion of cytoplasts from malignant cells (nucleus absent) with karyoplasts from normal cells (nucleus present). The important feature of their study was that the non-transformed and the transformed cells were all derived from an original cloned progenitor cell with a common nuclear and cytoplasmic background (Israel and Schaeffer, 1988; Seyfried, 2012d). These findings showed that normal cell nuclei could not suppress tumorigenesis when placed in tumor cell cytoplasm. In other words, normal nuclear gene expression, which would presumably include tumor suppressor genes, was unable to suppress malignancy. An alternative view is that the cytoplasm of the tumor cell could reprogram the nucleus to become tumorigenic. These findings are consistent with the view of Darlington who showed that it was the cytoplasm, rather than the nucleus, that determined the tumorigenic state of the cells (Darlington, 1948). Israel and Schaeffer did not identify the molecular basis for the cytoplasmic control of tumorigenesis, but they did suggest that epigenetic changes in nuclear gene expression might be responsible for the phenomenon (Seyfried, 2012d).

It is obvious that the findings of Israel and Schaeffer are inconsistent with the somatic mutation theory, but their observations would support the concepts of Warburg's theory. These investigators, however, did not connect their observations to Warburg's theory. Instead they linked their observations to a potential epigenetic phenomenon (Israel and Schaeffer, 1988). It is important to recognize that mitochondria are a powerful extra nuclear epigenetic system that can control nuclear gene expression through the retrograde signaling system (Minocherhomji et al., 2012; Seyfried, 2012e). A personal account of the Israel and Schaeffer studies in light of the competing theories of cancer has appeared (Christofferson, 2014).

The findings of Israel and Schaeffer that normal cytoplasm could suppress tumorigenicity were also consistent with the observations of Shay and Werbin (Shay and Werbin, 1988; Shay et al., 1988; Seyfried, 2012d). Shay and Werbin identified several factors that could influence the outcome of cybrid experiments designed to uncover cytoplasmic suppressors of tumorigenicity. These influencing factors included, (1) the relative amounts of non-tumorigenic and tumorigenic cytoplasm in cybrids; (2) the time interval that cybrids are passaged in culture prior to testing their tumorigenicity; (3) whether or not mutagenesis with carcinogens were used to introduce genetic markers in the cells; and (4) the type of cell combinations that were used. It would not therefore be surprising that varied results could occur with the cybrid experiments if the confounding variables were not controlled. In general, however, the observations of Shay and Werbin were consistent with the conclusion of the Israel and Schaeffer experiments. Although Shay and Werbin mentioned a possible role for mitochondria in the suppressive effects of the cytoplasm on tumorigenesis, they also did not consider their results in light of Warburg's metabolic theory (Seyfried, 2012d).

Howell and Sager, however, were aware of the relationship of Warburg's theory and the findings from the various cybrid studies (Howell and Sager, 1978; Seyfried, 2012d). These investigators speculated that the results from the cybrid experiments could help distinguish whether it was the cytoplasm or the nucleus or that determined tumorigenicity. They showed that cytoplasm of non-tumorigenic normal cells suppressed the rate and extent of tumor formation in nude mice when fused with nucleated tumorigenic counterparts (Seyfried, 2012d). Howell and Sager stated*; “if tumor cell mitochondria are defective, as Warburg postulated, then suppression could result from the introduction of mitochondria from normal cells into cybrids”* (Howell and Sager, 1978). These findings like those of Koura, Israel and Schaeffer, and Shay and Werbin supported Warburg's theory and are difficult to explain with the somatic mutation theory (Seyfried, 2012d).

To further evaluate the role of the cytoplasm and the nucleus in the control of malignancy, Jonasson and Harris conducted several interesting studies in human/mouse hybrids. These investigators evaluated *in vivo* tumor malignancy in a range of hybrid clones derived from fusions of a malignant mouse melanoma with diploid human fibroblasts and lymphocytes (Jonasson and Harris, 1977). They observed that the human diploid cells were as effective as the mouse diploid cells in suppressing the malignancy of the mouse melanoma cells, despite the preferential elimination of the human chromosomes in the hybrid clones. Malignancy was also suppressed in a hybrid clone where only a single human X chromosome was present. Jonasson and Harris showed that this clone continued to produce few tumors, even after they used back selection to remove this remaining X chromosome. These findings suggested that no human nuclear genetic material was responsible for suppression of malignancy. These findings would rule out a nuclear epigenetic explanation for suppression of tumorigenesis, but would not exclude an extra-nuclear (mitochondrial) epigenetic explanation.

Jonasson and Harris also constructed hybrids between the melanoma cells and human fibroblasts that were irradiated before cell fusion (Jonasson and Harris, 1977). They showed that the incidence of tumor take in nude mice was greater in crosses between the mouse melanoma cells and the irradiated human fibroblasts than in crosses between the melanoma cells and the un-irradiated human fibroblasts (Jonasson and Harris, 1977). These investigators concluded that the suppression of malignancy involved the participation of a radio-sensitive extra-chromosomal element. The findings from the Jonasson and Harris studies were interesting for several reasons (Seyfried, 2012d). First, their observations were consistent with those of several other cybrid studies suggesting that factors in normal cytoplasm could suppress tumorigenicity. Second, no human nuclear genetic material was responsible for the suppressive effect. Lastly, high-dose gamma radiation could destroy the cytoplasmic factor that was responsible for tumor suppression. This last observation was consistent with the findings of both Warburg and Darlington in showing that high-dose radiation destroys mitochondrial respiration and the cytoplasmic plasmagene, which has multiple characteristics of mitochondria (Darlington, 1948; Warburg, 1956a). Low dose radiation can cause nuclear mutations but not cancer, whereas high dose radiation damages both the nucleus and mitochondria and can cause cancer.

It is interesting that Jonasson and Harris excluded the mitochondria in preference to a centrosome origin for the suppression effect of cytoplasm on tumorigenesis (Jonasson and Harris, 1977). Their opinion was based largely on the findings of other investigators showing that no human mitochondrial DNA or proteins were detected in the human-mouse cybrids. More recent studies in transmissible cancers, however, show that tumor mitochondria can integrate with normal mitochondria in some tumors (Rebbeck et al., 2011). I suggested that this integration might reduce or partially correct the respiratory insufficiency in the tumor cell mitochondria thus suppressing tumorigenicity (Seyfried, 2012d). The work of King and Attardi also support this possibility in showing that exogenous mtDNA could enhance respiration in cells lacking functional mtDNA (King and Attardi, 1988, 1989). The more recent findings of Tan et al. also support the possibility in showing that mtDNA can be transferred horizontally from host cells to tumor cells in the microenvironment (Tan et al., 2015). Viewed collectively, these observations are in general agreement with Warburg's original theory.

It is not possible, however, to exclude all influence of the nuclear genome in the suppression of tumorigenicity. Saxon and co-workers showed that the microcell transfer of Chromosome 11 suppressed tumorigenicity in HeLa cells (Saxon et al., 1986). They suggested that a tumor suppressor gene could be present on Chromosome 11. These findings also suggest an interaction between Chromosome 11 and mitochondria (Seyfried, 2012d). Is it possible that a gene on Chromosome 11 facilitates mitochondrial respiration thus suppressing tumorigenicity in the HeLa cells? (Seyfried, 2012d). It is also interesting that defects on Chromosome 11 have been associated with the Wilms kidney tumor and with childhood neuroblastoma. Further studies will be needed to determine if tumorigenic suppression involves interactions between mitochondrial respiration and genes on Chromosome 11.

## Evidence from rho^0^ cells supporting a mitochondrial origin of tumorigenesis

Singh and co-workers showed that the exogenous transfer of wild type mitochondria to cells with depleted mitochondria DNA (rho^0^ cells) could reverse the altered expression of the APE1 DNA repair protein and the tumorigenic phenotype, thus providing evidence for the role of mitochondria in the suppression of tumorigenicity (Singh et al., 2005). Mitochondrial respiration appears responsible for the efficiency of APE1-mediated DNA repair. The rho^0^ cells have impaired respiration due to the lack mtDNA that is essential for normal cellular respiration. It is my view that transfer of normal mtDNA to the rho^0^ cells will restore respiration, turn off the mitochondria/nuclear retrograde response, and prevent nuclear genomic instability (Seyfried, 2012d). These findings suggest that it is efficient mitochondrial respiration that prevents cancer. The more recent studies of Cruz-Bermudez support these observations (Cruz-Bermúdez et al., 2015). I also described how mitochondrial enhancement therapies could prevent cancer (Seyfried, 2012b).

Wallace, and colleagues also provided support for the importance of respiration in the origin of prostate cancer (Petros et al., 2005). These investigators introduced the T8993G pathogenic mtDNA mutation into PC3 prostate cancer cells through cybrid transfer to determine whether the mutant pancreatic tumors expressed increased ROS levels and growth rate. The engineered PC3 prostate cancer cells were then tested for tumor growth in nude mice. The resulting mutant T8993G cybrids produced tumors that were seven-times larger than those produced from the wild-type cybrids. In contrast to the rapid growth of the mutant cybrids, the wild-type cybrids grew very slowly in the mice. Significantly more ROS were also produced in the tumors derived from T8993G mutant cybrids than tumors without this mutation. The carcinogenic and mutagenic action of ROS will damage respiration and produce nuclear genomic instability (Waris and Ahsan, 2006; Klaunig et al., 2010; Seoane et al., 2011). Additional experiments from the Wallace group and more recently from Cruz-Bermudez and co-workers showed that introduction of mtDNA mutations could reverse the anti-tumorigenic effect of normal mitochondria in cybrids (Petros et al., 2005; Cruz-Bermúdez et al., 2015). These findings indicate that some mtDNA mutations can play an important role in the etiology of cancer and that cancer can be best defined as a type of mitochondrial metabolic disease. These findings are also more in line with Warburg's theory than with the somatic mutation theory.

## Normal cytoplasm suppresses tumorigenesis *in vivo*: the lucke frog renal tumor

Substantial information exists showing that the nuclei of tumor cells can be reprogrammed to form normal tissues when they are transplanted into normal cytoplasm, despite the continued presence of the tumor-associated genomic defects in the cells of the derived tissues (Seyfried, 2012d). McKinnell, Deggins, and Labat showed that cell nuclei from frog renal tumors could direct normal frog development following transplantation of the renal tumor cell nucleus into an enucleated normal egg cell (McKinnell et al., 1969). The experiments involved implantation of nuclei, isolated from Lucke frog renal cell tumors, into fertilized enucleated eggs from normal diploid frogs. Importantly, the cells of the renal tumor were triploid in containing three copies of all chromosomes. Triploid tadpoles developed normally from the triploid tumor cell nuclei, and revealed functional tissues of many types. This experimental strategy made it possible to distinguish development initiated by the transplanted nucleus from development influenced by an inadvertently retained maternal diploid nucleus (McKinnell et al., 1969). “*The investigators showed that ciliated epithelium propelled the tadpoles in the culture dishes. The tadpoles swam when stimulated. The tadpoles had functional receptors, nerve tissue, and striated muscle necessary for swimming. Cardiac muscle pumped blood cells through the gills. Suckers secreted abundant mucus. Clearly seen were a pronephric ridge, eye anlage, nasal pit, and open mouth, as was the differentiation of the head, body, and the tail. The tail fin regenerated after being clipped for chromosome study. Moreover, sections of embryos developed from transplanted triploid tumor nuclei revealed apparent normal development of the brain, spinal cord, optic cup with lens, auditory vesicle, somites, pronephric tubules, pharynx, midgut, and notochord. No evidence of abnormal cell growth was seen in any of the organs or tissues examined*” (Seyfried, 2012d). These findings showed that nuclei derived from tumor cells could direct normal developmental and did not induce dysregulated cell growth, the signature phenotype of tumorigenesis. It is interesting that the tadpoles containing tumor nuclei could not complete development to normal adult frogs. It remains unclear if the tumor-associated nuclear defects were responsible for preventing late stage development of the frogs.

The findings from the Lucke frog experiments are consistent with the mitochondrial metabolic theory, but are difficult to reconcile with the somatic mutation theory of cancer (Seyfried, 2012d). The enucleated egg would contain the mitochondria from the normal egg cytoplasm. These mitochondria would direct normal energy homeostasis during development. It is my view that normal energy homeostasis “*suppresses tumorigenesis despite the presence of the tumor nucleus and somatic mutations*” (Seyfried, 2012d). Later studies suggested that loss of the Lucke tumor herpes virus was linked to the loss of tumorigenicity (Carlson et al., 1994). This virus was considered the etiological agent responsible for the origin of the renal tumors. It is now known, however, that the herpes virus can alter mitochondrial function to induce tumorigenesis (D'agostino et al., 2005; Seyfried, 2012c). Indeed, Ackerman and Kurtz showed that herpes viruses have an intimate attachment to mitochondria that causes dysfunctional respiration (Ackermann and Kurtz, 1952). Hence, the replacement of virus-damaged mitochondria with normal mitochondria from the host could the suppress tumorigenesis despite the presence of the renal tumor nucleus (Seyfried, 2012d). The findings from the frog renal tumor are similar to those described above from the cell cybrid experiments, and cast doubt on the somatic mutation theory as an explanation for this type of cancer.

## Normal cytoplasm can suppresses tumorigenic phenotypes in mice

**Figure 1:**
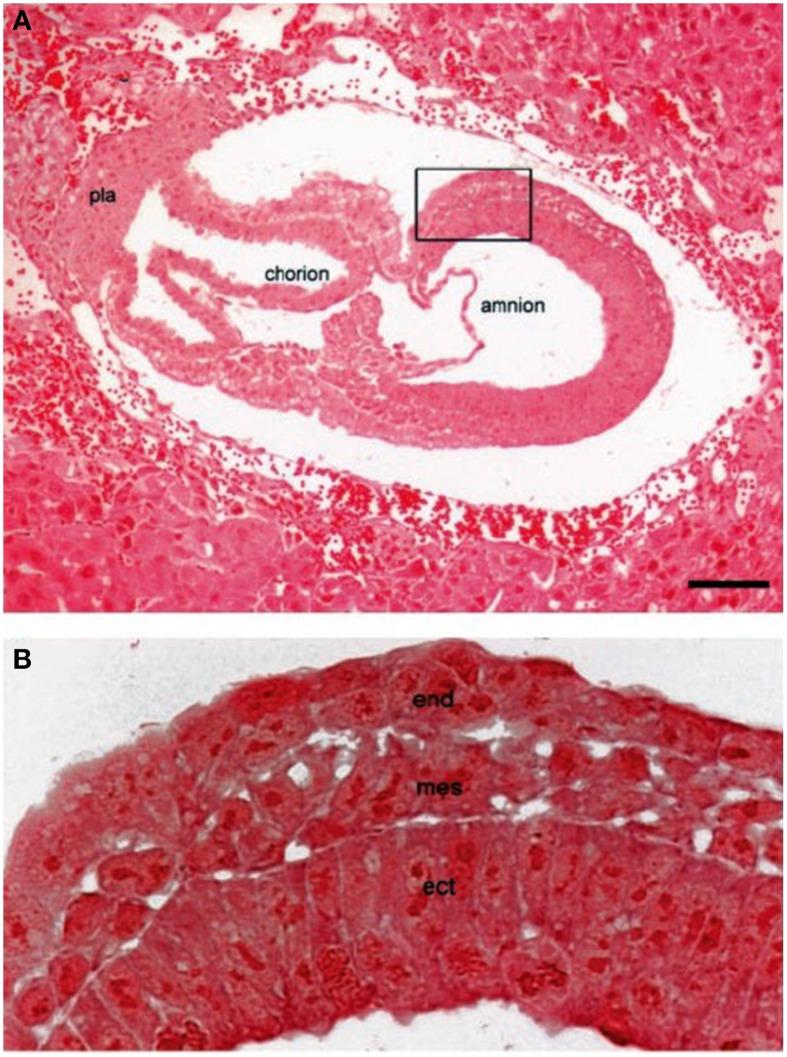
**Nuclei from brain tumors support normal mouse embryonic development. (A)** H&E staining of a mouse embryo (embryonic day, E-7.5) derived from a cell containing the transplanted “nucleus” from a medulloblastoma tumor. **(B)** the boxed area in **(A)** (at a higher magnification) showing the three germ layers; ecto-placental cone (pla); embryonic endoderm (end); embryonic mesoderm (mes,); embryonic ectoderm (ect), Scale bar, 20 μm. The cytoplasm will contain normal mitochondria. The results show that a nucleus derived from a brain tumor can direct normal embryonic development when implanted into normal cytoplasm. Reprinted with permission from Li et al. (2003).

Findings similar to those obtained with the Lucke frog renal tumor were also obtained following nuclear transfer in mouse tumors. Morgan and colleagues showed that nuclei from a mouse brain tumor, arising from cerebellar granule cells (medulloblastoma), could direct normal development when the tumor nuclei were transplanted into enucleated somatic cells (Li et al., 2003). Figure 1 from their study shows that normal embryonic tissues and germ cell layers can be formed from cells containing the tumor nuclei. These investigators showed that the transfer of the tumor cell nucleus into normal cytoplasm suppressed the tumorigenic phenotype despite the continued presence of the mutant nuclear gene (*Patched*) that was thought responsible for the original tumorigenic phenotype (Li et al., 2003; Seyfried, 2012d). The transplanted medulloblastoma nuclei produced post-implantation embryos that underwent normal tissue differentiation and early stage organogenesis. Importantly, no malignancies or abnormal cell growth were seen in any of the recipient mice. Normal proliferation control was observed in cultured blastocysts indicating that nuclear somatic mutations alone were not likely responsible for the original tumorigenic phenotype (Li et al., 2003).

Li and co-workers suggested that the tumorigenic *Patched* mutation causing medulloblastoma must act within the context of the cerebellar granule cell lineage, and that the mutation did not support the malignant cell proliferation outside the cerebellum (Li et al., 2003). Although an epigenetic reprogramming of the medulloblastoma nuclei was offered as an explanation for their observations, it is also possible that their observations resulted from the replacement of dysfunctional mitochondria with normally functional mitochondria that would be present in the recipient stem cell (Seyfried, 2012d). The results in this mouse brain tumor model are consistent with those seen in the Lucke frog renal tumor. The findings from Li et al would also support the earlier observations of Mintz and Illmensee showing that normal appearing mice could be cloned from tumor cell nuclei obtained from malignant teratomas, and that “*structural mutations in the nuclear genome could not be responsible for tumor formation”* (Mintz and Illmensee, 1975). Considered collectively, these findings indicate that nuclear gene mutations alone cannot account for the origin of tumors. Although the observations reveal the potential role of mitochondria in modulating tumorigenesis, they are difficult to explain under the somatic mutation theory.

**Figure 2:**
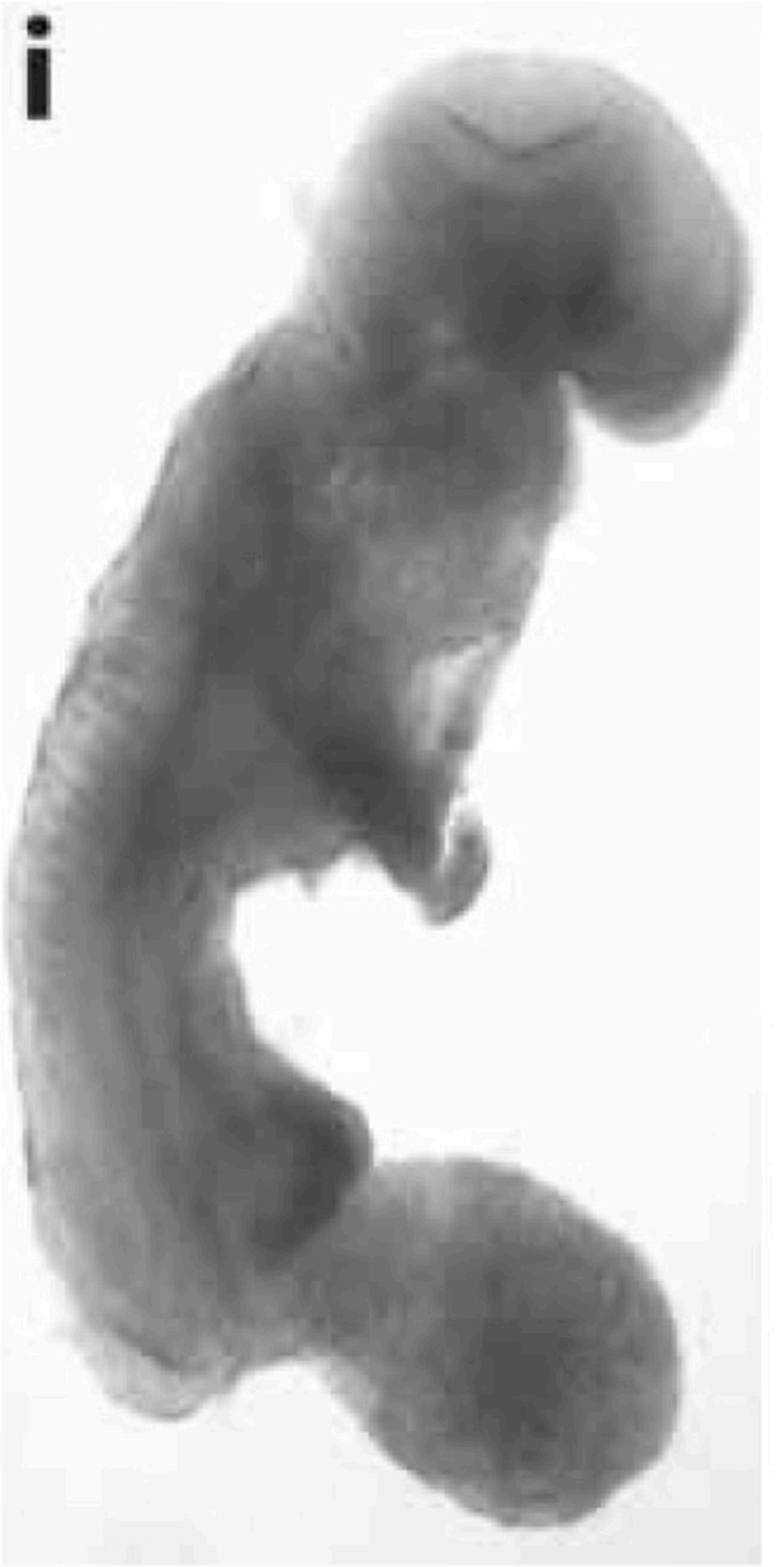
**Mouse embryo cloned from tumor cell nucleus**. An E-9.5 mouse embryo cloned from a melanoma-derived R545-1 embryonic stem cell. The embryo expressed neural tube closure, a beating heart, and normal limb bud development consistent with regulated cell growth. The result shows that the nucleus of a malignant melanoma can direct early mouse development when placed into normal cytoplasm containing normal mitochondria. However, irreversible genetic alterations, from the melanoma donor genome, disrupted complete development similar to the situation found in Lucke frogs that were cloned from nuclei of renal tumors (McKinnell et al., 1969). Reprinted from in this figure (Hochedlinger et al., 2004) with permission.

The work of Hochedlinger, Jaenisch, and colleagues also supported the findings from the Lucke frog and the mouse medulloblastoma experiments (Hochedlinger et al., 2004). These investigators found that the nuclei of mouse melanoma cells could produce normal-appearing blastocysts without signs of dysregulated cell proliferation. They also showed that normal blastocysts could be formed from *p53* -/- breast cancer cells, and that normal blastocysts and embryonic cell lines could be formed from melanoma nuclei. Figure 2 shows an image from their study of mouse embryo cloned from the nucleus of a melanoma. These investigators suggested that the oocyte environment could suppress the malignant phenotype of the various tumor types, and that tumor nuclei could direct normal appearing development in early mouse embryos (Hochedlinger et al., 2004; Seyfried, 2012d). The oocyte cytoplasm would be expected to contain normal mitochondria. Based on the previously mentioned studies in cybrids, frogs, and mice, it would be reasonable to assume that the respiratory competent normal mitochondria would suppress tumorigenicity. It can be suggested that tumor nuclei would direct normal development as long as normal functioning mitochondria exist in the cytoplasm. However, the authors showed that tumors could form in some mice cloned from tumor nuclei, as long as the *Ras* oncogene was expressed together with the tumor-associated mutations. It is now known that the *Ras* oncogene induces tumorigenesis through an inhibitory effect on mitochondrial oxidative phosphorylation (Hu et al., 2012). Hence, respiratory damage is an essential requirement for tumorigenesis.

The studies from the Hochedlinger and Jaenisch group also showed that embryonic stem cells, derived from cloned melanoma cells, could differentiate into multiple somatic cell lineages including fibroblasts, lymphocytes, and melanocytes (Hochedlinger et al., 2004). “*Remarkably, normal development occurred despite the persistence of severe chromosomal changes and mutations documented by array-comparative genome hybridization (CHG)”* (Hochedlinger et al., 2004; Seyfried, 2012d). *The investigators concluded that secondary chromosomal changes*, associated with malignancy, *do not necessarily interfere with pre-implantation development, embryonic stem cell derivation, and a broad nuclear differentiation potential* (Hochedlinger et al., 2004). These observations suggest that nuclear gene mutations alone cannot account for the origin of cancer and are therefore inconsistent with the somatic mutation theory. Unfortunately, these investigators also did not discuss their findings in relationship to mitochondrial function or to the Warburg theory despite showing evidence in line with the theory.

## Suppressed tumorigenicity in the liver microenvironment

Grishman and co-workers reported that rat liver tumor cell lines expressing aneuploidy formed aggressive tumors when grown subcutaneously, but did not form tumors when grown orthotopically in the liver (Coleman et al., 1993; Seyfried, 2012d). The tumor cells became morphologically differentiated following intrahepatic transplantation in adult syngeneic Fischer 344 rats. The authors concluded that close cell contacts or factors in the hepatic microenvironment suppressed tumorigenicity. It is known that cell-cell fusions occur in murine liver (Faggioli et al., 2008). Could it be possible that fusion between normal hepatic cells and neoplastic hepatic cells, in the unique liver microenvironment, might suppress tumorigenicity in a manner similar to that seen in the cybrid experiments mentions above? The recent findings from Tan and colleagues support the horizontal transfer of mitochondrial DNA from host cells to tumor cells (Tan et al., 2015). Further studies will be needed to resolve issues of horizontal transfer of mitochondrial components in the liver microenvironment.

The suppressive effect of normal mitochondria on tumorigenesis links mitochondrial function to the long-standing controversy on cellular differentiation and tumorigenicity (Harris, 1988; Soto and Sonnenschein, 2004; Seyfried, 2012d; Seyfried and Shelton, 2010). *Respiration is required for the emergence and maintenance of differentiation, while loss of respiration leads to glycolysis, dedifferentiation, and unbridled proliferation* (Seyfried, 2012d). This observation is consistent with the general hypothesis presented in this review, that prolonged impairment of mitochondrial energy metabolism underlies carcinogenesis (Warburg, 1969; Szent-Gyorgyi, 1977; Seyfried, 2012d). This hypothesis would represent an epigenetic origin of the disease in the classic sense (Nanney, 1958; Holliday, 2006; Seyfried, 2012d). Replacement of dysfunctional mitochondria with normal mitochondria will restore normal energy homeostasis and the differentiated state.

## Normal mitochondria can suppress tumorigenesis in metastatic breast cancer

Recent studies from Kaipparettu, Wong, and colleagues show that the introduction of non-cancerous mitochondria into highly malignant breast cancer cells could reverse malignancy and down regulate several oncogenic pathways, including those involved with unregulated cell growth, viability under hypoxia, anti-apoptotic properties, resistance to anti-cancer drug, invasion, colony formation in soft agar, and *in vivo* tumor growth in nude mice (Kaipparettu et al., 2013). Cybrids with normal mitochondria showed enhanced mitochondrial function including increased ATP synthesis, oxygen consumption and respiratory chain activities despite the presence of the cancerous nuclear genome. A remarkable finding was that even though genes that encode most mitochondrial proteins are located in the nucleus, introduction of mitochondria derived from the non-cancerous cell to a cancer nuclear environment resulted in suppression of oncogenic pathways and the tumorigenic phenotype. Cruz-Bermudez et al recently reported similar findings in showing that *in vivo* tumorigenicity was significantly lower in cybrids containing the 143B osteosarcoma cell nucleus and normal mitochondria than in 143B cells containing mitochondria harboring various mutations in the mtDNA (Cruz-Bermúdez et al., 2015). In other words, normal mitochondria could suppress tumorigenicity despite the continued presence of the tumorigenic nucleus. The results from these studies complement those from the above mentioned nuclear transfer experiments and highlight the important role of mitochondria in the origin and regulation of tumorigenesis. These findings are in line with the view that tumorigenesis arises more from mitochondrial defects than from somatic mutations in the nuclear genome.

## Inconsistencies and difficulties

Any theory that attempts to explain a complex biological phenomenon like cancer should address difficulties or inconsistencies with the theory, rather than ignore them. I have attempted to address these issues. For example, Akimoto, Hayashi, and co-workers reported that genome chimera mouse fibroblasts carrying nuclear DNA from tumor cells and mtDNA from normal cells expressed tumorigenicity, whereas those carrying nuclear DNA from normal cells and mtDNA from tumor cells did not (Akimoto et al., 2005). These observations suggest that nuclear DNA, but not mtDNA, was responsible for carcinogen-induced malignant transformation in these mouse fibroblasts. These findings raise an issue regarding the role of the nucleus and mitochondria in the origin of tumors and should be considered in light of the *in vivo* nuclear transfer experiments reported in this review. Israel and Schaeffer described the role of diverse *in vitro* histories in contributing to some inconsistencies seen in the cybrid studies (Israel and Schaeffer, 1988). Further studies will be needed to reconcile differences in results obtained from some *in vivo* and *in vitro* transfer experiments.

However, the role of mitochondrial DNA (mtDNA) in the origin and progression of cancer is controversial. We were unable to find any pathogenic mtDNA mutations in a broad range of chemically induced and naturally arising mouse brain tumors (Kiebish and Seyfried, 2005). Our studies were comprehensive in that we sequenced the entire mitochondrial genome after first isolating and purifying the mitochondria from the tumor tissue. Many of the reported mtDNA mutations found in tumors are thought to arise as artifacts possibly through amplification of nuclear embedded mtDNA sequences (NUMTs) (Salas et al., 2005; Schon et al., 2012). On the other hand, the tumorigenic phenotype is associated with abnormal mitochondrial lipids (Kiebish et al., 2008). Indeed, no tumor has yet been found with a normal content or composition of cardiolipin, the signature lipid of the inner mitochondrial membrane that regulates oxidative phosphorylation (Kiebish et al., 2009; Claypool and Koehler, 2012; Seyfried et al., 2014). Proteomic abnormalities involving mitochondria have also been reported in various tumors (Unwin et al., 2003; Ristow and Cuezva, 2009; Dai et al., 2010; Deighton et al., 2014). These findings provide additional evidence in support of Warburg's original theory. Pedersen documented the broad range of mitochondrial abnormalities that are found in tumor cells (Pedersen, 1978). Hence, mitochondrial abnormalities linked to cancer can involve more than just mtDNA mutations. We recently summarized how most cancers can arise from abnormalities in mitochondria structure and function (Seyfried, 2012a; Seyfried et al., 2014).

If most cancers arise from chronic abnormalities in mitochondrial respiratory capacity, why would cancer be rare in some persons that inherit mutations damaging mitochondrial function? For example, cancer is rare in patients with familial amyotrophic lateral sclerosis (ALS) (Vigliani et al., 2000). Familial ALS involves mutations in the Cu/Zn superoxide dismutase gene (SOD), which disturbs respiratory function and leads to neurodegeneration (Rosen, 1993; Dupuis et al., 2004a,b). Tumors arising in neurons of the central nervous system are rare, however, due to the inability of neurons to sustain fermentation when respiration is compromised (Allen et al., 2005). For example, mitochondrial ROS kills dopaminergic neurons in Parkinson's disease without producing cancer (Eng et al., 2003). Cancer is also rare in children with Barth syndrome that involves abnormalities in cardiolipin remodeling and respiratory dysfunction (Claypool and Koehler, 2012; Clarke et al., 2013). Children with Barth syndrome, however, also express hypoglycemia, which would impede glucose fermentation and the Warburg effect that would be needed to drive tumorigenesis. Most tumors arise in cells that can up-regulate the glycolytic pathway in order to compensate for a gradual and chronic disruption in oxidative phosphorylation. Cells that cannot make the energy transition from respiration to fermentation will die and never become tumorigenic, as Warburg first mentioned (Warburg, 1956a).

Multiple symmetric lipoma tumors with abnormal mitochondria were found in carriers of the inherited mitochondrial syndrome, myoclonus epilepsy and ragged-red fibers (MERRF) (Holme et al., 1993). The lipomas expressed mutations in the mtDNA gene encoding tRNA-lysine indicating that the lipomas arose from the mtDNA mutations. The mtDNA mutations were also linked to nuclear genomic instability involving gross chromosomal abnormalities. Although the tumors arose from the mtDNA mutation, the nuclear genomic instability could have arisen as a secondary consequence of the reported mitochondrial abnormalities. These findings would be consistent with the view that the genomic instability seen in tumors results as a secondary downstream effect of mitochondrial dysfunction and altered oxidative phosphorylation (Seyfried and Shelton, 2010; Chandra and Singh, 2011; Seyfried et al., 2014; Bartesaghi et al., 2015). Recent findings from Cruz-Burmedez et al show that tumorigenicity is greater in association with mtDNA mutations that provoke less severe mitochondrial dysfunction then with mtDNA mutations that provoke severe dysfunction in oxidative phosphorylation (Cruz-Bermúdez et al., 2015). These findings suggest that some level of mitochondrial oxidative phosphorylation is required for tumor initiation. However, oxidative phosphorylation is not likely necessary for progression of highly malignant breast tumors that have few if any mitochondria (Elliott et al., 2012). I agree with the view of Eng and colleagues that further research is needed into the genetic, cellular, and clinical aspects of mitochondrial function in relationship to cancer risk (Eng et al., 2003).

## Summary of nuclear-cytoplasmic transfer experiments

Considered collectively, the findings reviewed here provide compelling evidence showing that nuclear somatic mutations alone cannot account for the origin of tumors, and that normal cytoplasm containing mitochondria can suppress tumorigenicity. It is interesting that the findings from the nuclear-cytoplasmic transfer experiments are generally consistent across a broad range of tumor types, animal species, and experimental techniques. Several leaders in the field of genetics and developmental biology conducted these studies (C. D. Darlington, H. Harris, B. Mintz, R. Sager, J. Morgan, R. Jaenisch), which further supports the validity of the findings. Moreover, most of the studies were not done to test the somatic mutation theory of cancer, but rather were done to determine the importance of nuclear mutations in directing the tumorigenic phenotype. Consequently, data interpretation was largely unbiased. The general reproducibility of the findings is notable in light of recent concerns regarding the irreproducibility of important scientific results (McNutt, 2014). Although numerous inconsistencies have been documented that undermine the credibility of the somatic mutation theory (Sonnenschein and Soto, 2008; Burgio and Migliore, 2015), none of these are as powerful as those presented here from the nuclear-cytoplasmic transfer experiments. Moreover, recent studies from Chernet and Levin have shown that alterations in bioelectric membrane signaling can produce metastatic behavior of *Xenopus* melanocytes in the absence of somatic mutations further suggesting that the tumorigenic phenotype is not dependent on nuclear gene mutations (Chernet and Levin, 2014; Chernet et al., 2014). In other words, nuclear mutations alone are insufficient for producing tumors, whereas the tumorigenic phenotype can be produced in some cells without nuclear mutations. These findings seriously question the foundation of the somatic mutation theory of cancer.

**Figure 3:**
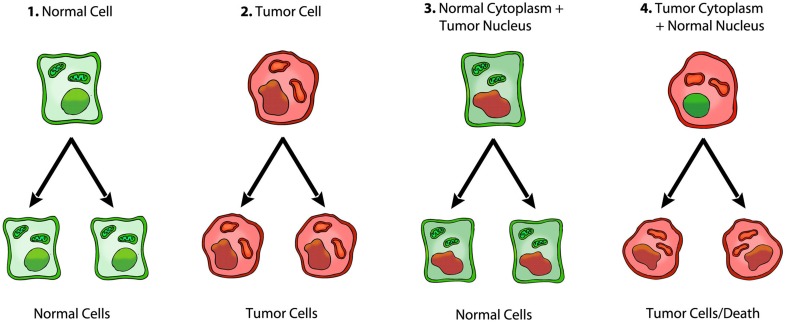
**Role of the nucleus and mitochondria in the origin of tumors**. Summary of a role of the mitochondria in the origin of tumorigenesis, as we previously described (Seyfried, 2012d; Seyfried et al., 2014). Normal cells are shown in green with nuclear and mitochondrial morphology indicative of normal gene expression and respiration, respectively. Tumor cells are shown in red with abnormal nuclear and mitochondrial morphology indicative of genomic instability and abnormal respiration, respectively. “(*1) Normal cells beget normal cells. (2) Tumor cells beget tumor cells. (3) Transfer of a tumor cell nucleus into a normal cytoplasm begets normal cells, despite the presence of the tumor-associated genomic abnormalities. (4) Transfer of a normal cell nucleus into a tumor cell cytoplasm begets dead cells or tumor cells, but not normal cells. The results suggest that nuclear genomic defects alone cannot account for the origin of tumors, and that normal mitochondria can suppress tumorigenesis*” (Seyfried, 2012d). Original diagram from Jeffrey Ling and Thomas N. Seyfried, with permission.

Although the nuclear-cytoplasmic transfer experiments fail to support the somatic mutation theory, the data from these experiments strongly support the Warburg theory of cancer. Normal mitochondrial function reverses expression and the Warburg effect because this effect is due to insufficient respiration (Burk and Schade, 1956; Kaipparettu et al., 2013; Seyfried et al., 2014). Aerobic fermentation is an effect of insufficient respiration. Statements about a “reverse Warburg effect,” which do not involve restored respiration in the tumor cells, are difficult to reconcile in light of the information presented here (Pavlides et al., 2009; Seyfried, 2012d). Normal mitochondria would enhance respiration thus suppressing oncogene expression and tumorigenicity, whereas mitochondria taken from cancer cells cannot restore respiration or suppress tumorigenicity. According to Warburg's theory, it would be expected that the presence of normal mitochondria in tumor cells would restore the cellular redox state, down regulate the mitochondrial stress response, and ultimately reduce or eliminate the need for fermentation (the Warburg effect) to maintain viability (Seyfried et al., 2014). In rephrasing, *normal mitochondrial function maintains the differentiated state thereby suppressing carcinogenesis, whereas dysfunctional mitochondria can enhance cellular dedifferentiation thereby facilitating carcinogenesis* (Seyfried, 2012d). Cuezva and Ristow also show that normal mitochondrial respiration suppresses tumorigenesis (Ristow, 2006; Cuezva et al., 2009; Ristow and Cuezva, 2009). Proliferation is the default state of metazoan cells, i.e., the state under which cells are found when they are freed from any active control (Sonnenschein and Soto, 1999). Mitochondria can maintain the differentiated state and quiescence. The loss of mitochondrial function will lead eventually to the default state of unbridled proliferation, i.e., the metabolic phenotype that was present in all cells during the anoxic alpha period of earth's history (Szent-Gyorgyi, 1977; Seyfried, 2012a). Figure 3 summarizes the role of mitochondria in tumorigenesis.

## The origin of cancer

It is well-known that reactive oxygen species (ROS) are produced in defective mitochondria largely through the coenzyme Q couple (Veech, 2004). ROS are powerful mutagens of nuclear DNA and can cause the genomic instability seen in most tumor cells (Waris and Ahsan, 2006; Seoane et al., 2011). Bartesaghi et al recently showed that nuclear genomic instability, p53 inactivation, and tumorigenic transformation occurred in neural progenitor cells following damage to their oxidative phosphorylation (Bartesaghi et al., 2015). It has been my view that the plethora of random somatic mutations seen in tumors of almost every kind arise ultimately as downstream effects of insufficient respiration with compensatory fermentation (Seyfried and Shelton, 2010; Seyfried, 2012a). Evidence is now accumulating in support for this view (Waris and Ahsan, 2006; Seoane et al., 2011; Al Mamun et al., 2012; Bartesaghi et al., 2015). Indeed, the gradual transformation in cellular energy production from oxidative phosphorylation to substrate level phosphorylation can account not only for the collection of random mutations and genomic instability seen in cancer cells, but can also account for all of the disease hallmarks described by Hanahan and Weinberg (Seyfried and Shelton, 2010; Hanahan and Weinberg, 2011; Seyfried, 2012a). We also showed that the hallmark of metastasis arises from damage to the respiration in cells of myeloid origin or their fusion hybrids, which would naturally possess the capability of surviving in the circulation and disseminating throughout the body (Huysentruyt and Seyfried, 2010; Seyfried and Huysentruyt, 2013). The data supporting the origin of cancer as a mitochondrial metabolic disease should be compared with the data supporting the “bad luck” origin of cancer through the somatic mutation theory (Tomasetti and Vogelstein, 2015). It should be obvious to most cancer biologists that the origin of cancer as a type of mitochondrial metabolic disease can explain better the hallmarks of the disease than can the somatic mutation theory.

The mechanism by which a broad range of disparate environmental carcinogens and rare germ line mutations might produce tumors through a common mechanism was referred to as the *oncogenic paradox* (Szent-Gyorgyi, 1977; Cairns, 1981; Seyfried et al., 2014). We recently explained the oncogenic paradox by describing how most, if not all, recognized carcinogens could damage cellular respiration thus shifting energy production from oxidative phosphorylation to substrate level phosphorylation (Seyfried and Shelton, 2010; Seyfried, 2012a; Seyfried et al., 2014). This would also include alterations in the tissue morphogenetic fields. A protracted shift from respiration to fermentation will acidify and destabilize the tissue microenvironment, and thus the morphogenetic field (Sonnenschein and Soto, 1999; Fosslien, 2008; Bissell and Hines, 2011). Microenvironment acidification enhances angiogenesis and facilitates the path to tumorigenesis (Sonnenschein and Soto, 1999; Gatenby and Gillies, 2004; Soto and Sonnenschein, 2004). This is consistent with view of the tumor as an unhealed wound (Dvorak, 1986). While many genetic abnormalities will arise through epigenetic phenomena, once established, genome instability could contribute to further respiratory impairment, genome mutability, and tumor progression (Rubin, 1985; Seyfried, 2001; Seyfried and Shelton, 2010). At some point, the nuclear genomic instability in the tumor cells would prevent a return to normal cellular homeostasis. What develops then is an escalating situation of biological chaos, where the intrinsic properties of the immune system (macrophages and local stroma) to heal wounds would enhance proliferation in tissue stem cells and their progenitors (Seyfried, 2001). Genomic instability and transformation accompany the biological chaos. Collectively, these powerful intrinsic properties drive each other to greater levels of biological disorder and unpredictability all of which arise initially from chronic injury to cellular respiration.

## Conclusion

In summary, the information presented here supports the notion that cancer originates from damage to the *mitochondria* in the cytoplasm rather than from damage to the *genome* in the nucleus. The genomic damage in tumor cells follows, rather than precedes, the disturbances in cellular respiration. This view is also consistent with the previous findings of Roskelley et al. (1943), Hu et al. (2012). It is unclear how many researchers in the cancer field are aware of the evidence supporting the mitochondrial origin of the disease. Payton Rous stated that; “*the somatic mutation theory acts like a tranquilizer on those who believe in it*” (Rous, 1959). Rous' statement was prophetic in light of the present embrace of the somatic mutation theory, despite the glaring inconsistencies with this theory. I attribute the slow progress in the “War on Cancer” to the persistent embrace of the somatic mutation theory, and to the failure in recognizing mitochondrial dysfunction as a credible alternative explanation for the origin of the disease (Seyfried, 2012a). We recently described how the somatic mutations in tumors cells would reduce adaptability to stress, thus making the tumor cells vulnerable to elimination through “press-pulse” metabolic therapies involving non-toxic drugs and ketogenic diets (Seyfried and Mukherjee, 2005; Seyfried et al., 2014). It is my opinion that real progress in cancer management and prevention will emerge once the cancer field abandons the somatic mutation theory and comes to recognize the role of the mitochondria in the origin, management, and prevention of the disease.

### Conflict of interest statement

The information presented is the view of Dr. Thomas N. Seyfried based on previous data that was published in peer-reviewed journals. The author declares that the research was conducted in the absence of any commercial or financial relationships that could be construed as a potential conflict of interest.
